# Intracellular Crosslinking of Filoviral Nucleoproteins with Xintrabodies Restricts Viral Packaging

**DOI:** 10.3389/fimmu.2017.01197

**Published:** 2017-09-27

**Authors:** Tamarand Lee Darling, Laura Jo Sherwood, Andrew Hayhurst

**Affiliations:** ^1^Department of Virology and Immunology, Texas Biomedical Research Institute, San Antonio, TX, United States; ^2^Department of Microbiology, Immunology and Molecular Genetics, The University of Texas Health Science Center at San Antonio, San Antonio, TX, United States

**Keywords:** single-domain antibodies, VHH, Marburg, Ebola, nucleoprotein, crosslinker, intrabody, virus-like particle

## Abstract

Viruses assemble large macromolecular repeat structures that become part of the infectious particles or virions. Ribonucleocapsids (RNCs) of negative strand RNA viruses are a prime example where repetition of nucleoprotein (NP) along the genome creates a core polymeric helical scaffold that accommodates other nucleocapsid proteins including viral polymerase. The RNCs are transported through the cytosol for packaging into virions through association with viral matrix proteins at cell membranes. We hypothesized that RNC would be ideal targets for crosslinkers engineered to promote aberrant protein–protein interactions, thereby blocking their orderly transport and packaging. Previously, we had generated single-domain antibodies (sdAbs) against Filoviruses that have all targeted highly conserved C-terminal regions of NP known to be repetitively exposed along the length of the RNCs of *Marburgvirus* (MARV) and *Ebolavirus* (EBOV). Our crosslinker design consisted of dimeric sdAb expressed intracellularly, which we call Xintrabodies (X- for crosslinking). Electron microscopy of purified NP polymers incubated with purified sdAb constructs showed NP aggregation occurred in a genus-specific manner with dimeric and not monomeric sdAb. A virus-like particle (VLP) assay was used for initial evaluation where we found that dimeric sdAb inhibited NP incorporation into VP40-based VLPs whereas monomeric sdAb did not. Inhibition of NP packaging was genus specific. Confocal microscopy revealed dimeric sdAb was diffuse when expressed alone but focused on pools of NP when the two were coexpressed, while monomeric sdAb showed ambivalent partition. Infection of stable Vero cell lines expressing dimeric sdAb specific for either MARV or EBOV NP resulted in smaller plaques and reduced progeny of cognate virus relative to wild-type Vero cells. Though the impact was marginal at later time-points, the collective data suggest that viral replication can be reduced by crosslinking intracellular NP using relatively small amounts of dimeric sdAb to restrict NP packaging. The stoichiometry and ease of application of the approach would likely benefit from transitioning away from intracellular expression of crosslinking sdAb to exogenous delivery of antibody. By retuning sdAb specificity, the approach of crosslinking highly conserved regions of assembly critical proteins may well be applicable to inhibiting replication processes of a broad spectrum of viruses.

## Introduction

The idea of turning the humoral immune system inside out (1) as a means of intracellular immunization (2) was first demonstrated by Antman and Livingston in 1980 (3) where IgG specific for SV40 T antigens were capable of inhibiting viral DNA synthesis following microinjection into cells permissive for replication. Since that time, many approaches have been tried to deliver antiviral antibodies into the cell cytoplasm in a more efficient manner to transition the approach from experimental to therapeutic. An early validation step in this process is the intracellular expression of antibodies from transfected plasmid DNA that enables the rapid evaluation of the resulting “intrabodies.” The process is straightforward and enables screening for desired characteristics such as improved solubility and characterizing inhibitory activities. Rarely IgG genes have been directly employed in this approach for antiviral strategies (4) since they are complex multi-domain (*n* = 12) and multi-chain (*n* = 2) molecules tending to favor secreted environments for productive expression. Instead, smaller antibody fragment genes like Fab (four domains), scFv (two domains), and derivatives have been explored and shown to be functional within the reducing cytosol to varying degrees [for reviews, see Ref. (5, 6)].

Derived from heavy chain only antibodies of camelids, single-domain antibodies (sdAbs or VHH) (7) have also shown promise as intrabodies as they are one domain and one chain, not requiring pairing with a variable light-chain domain to bind antigen. sdAbs are highly soluble and are heat stable, which makes production at physiological temperatures more feasible. sdAbs are also generally not dependent on the formation of their intra-domain disulfide bond for productive expression, making them ideal candidates for expression in the reducing environment of the cytosol. Other small scaffolds have also shown promise as intrabody mimics by combining the simplicity of a single-domain unit that is disulfide bond free with engineered diversity for repertoire selection [e.g., the fibronectin fold (8, 9)].

Although all of these smaller derivatives have favorable production characteristics, they lack the steric bulk that can be advantageous for antiviral activity in occluding the interaction of viral proteins with each other or with host proteins. Since intrabodies are by definition within the cell, they also lack classical effector regions to enhance antiviral activity through classical antibody-dependent cell-mediated cytotoxicity. Unengineered sdAbs also lack the bivalency of IgG and are devoid of antiviral enhancements possible through avidity. Consequently, approaches to employ monovalent intrabodies and antibody mimetics as antivirals have tended to focus on impeding very specific viral functions where the impact of direct binding to target antigen is likely to be large. Examples include a scFv that binds to HPV16 E6 protein and inhibits p53 degradation (10) and a sdAb that inhibits HIV rev multimerization (11).

Selecting the right intrabodies to perform these roles for antiviral development is not trivial and requires identification of appropriate candidates from panels of pre-existing clones that not only express well but also have the desired inhibitory function. Alternatively, intrabody selection campaigns can be introduced to enrich high-expressing clones for screening of inhibitory function (12). More recently, direct selection of clones that allow cell survival after virus challenge with cytotoxic viruses can identify clones with antiviral activity (13).

Here, we advance an alternative approach based on a combination of rational yet simplistic design, basic antibody engineering principles, and a David versus Goliath mindset. We hypothesized that leveraging the steric bulk of macromolecular viral assemblies against one another would convert a normally innocuous monovalent sdAb into one with high-antiviral potency. Therefore, rather than focusing on *inhibiting* specific interactions between virus–virus or virus–cell proteins, we reasoned it should be possible to disrupt viral replication by *promoting* aberrant interactions. We aimed to crosslink cytosolic viral macromolecules using sdAb engineered as tandem dimers. In this manner, we should elicit a large impact on viral replication with a small amount of sdAb, which is ideal for advancing down a therapeutic track where high efficacy is ultimately required. We have christened these sdAb “Xintrabodies” to fuse the abbreviation for crosslinking (X) with the term for intrabody.

We had previously isolated sdAb from our semisynthetic llama library by live panning on *Marburgvirus* (MARV) (14) and *Ebolavirus* (EBOV) (15) at biosafety level four (BSL-4) which bound the C-terminal region of nucleoprotein (NP). All sdAb were capable of forming highly sensitive monoclonal affinity reagent sandwich assays (16) by reacting with detergent-treated virus preparations or recombinant NP suggesting the epitope they bound was displayed polyvalently along the NP polymer as visualized previously by others (17, 18). While our original plan was to employ these sdAbs in developing preclinical diagnostics, we rationalized they might also be promising candidates for exploring our crosslinking approach since the mass of NP polymers would be tens of MDa versus 30 kDa for the sdAb dimers.

**Figure 1:**
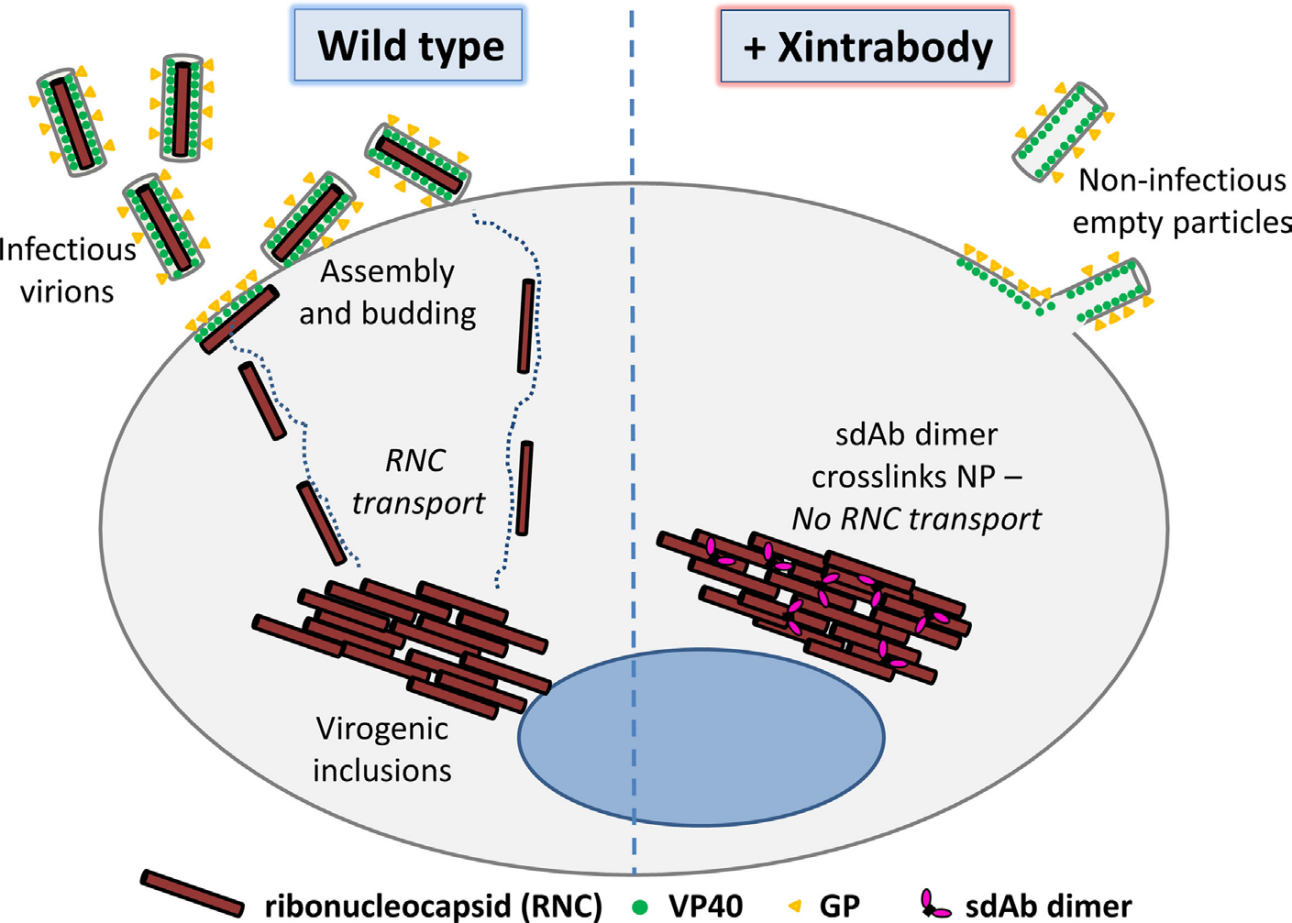
Overall hypothesis of the approach showing that crosslinking a viral structural protein within the cell using a multimeric single-domain antibody (sdAb) will impede viral replication by disrupting the orderly assembly of infectious virus particles (virions). The dimeric sdAb can be introduced into the uninfected target cell or virus-infected cell *via* endogenous gene expression, gene delivery, protein transfection, or protein transduction to mediate the antiviral activity. Here, we focus on utilizing *Marburgvirus* and *Ebolavirus* nucleoprotein (NP) as our model target since we have sdAb to hand that bind polyvalent assemblies. Our working theory is that our sdAb dimers or Xintrabodies are crosslinking NP epitopes among ribonucleocapsids (RNCs) within viral factories and inhibiting their transport to the cell membrane for further assembly into virions. It should be noted that other antigens involved in assembly may be equally effective provided suitable sdAb are available, although targeting antigens that occur in viral factories may be advantageous owing to high local concentrations. The overall approach may be applicable to many other viruses and scaffolding components and leverages relatively small amounts of minute 30 kDa dimeric sdAb crosslinking MDa sized targets to impede productive viral replication.

A peculiar feature of many viral replication pathways is the formation of virogenic inclusion bodies or virus factories that could lend themselves to being particularly attractive sinks for intrabodies. The high concentration of target antigens and compartmentalization of certain cell processes are thought to drive more efficient genome replication, viral component, and/or even viral particle assembly (depending on the particular virus). Consequently, these sites could be very vulnerable to a crosslinking strategy as opposed to targeting diffusely distributed antigens throughout the cytoplasm. For both MARV and EBOV, the inclusions are highly dynamic sites of replication and contain large numbers of NP polymers (19–21) and several other viral proteins (L, VP24, VP30, and VP35) that together form the ribonucleocapsid (RNC) that encapsidates the RNA genome. These RNC assemblies have been shown to leave the inclusions on a one by one basis for transport through the cytoplasm for assembly at the cell periphery (22, 23). At the membrane, the RNCs interact with matrix protein VP40 to form enveloped infectious virus particles studded with the host cell targeting molecule GP that are then released. We hypothesized that the introduction of Xintrabodies into this model system will crosslink the RNC within the inclusions and impede the migration of NP to the cell periphery and restrict its ability to be packaged (Figure 1).

Herein, we describe our studies on assembling, producing, and evaluating sdAb monomers and dimers *in vitro* and cell culture, comparing and contrasting their impacts on NP crosslinking *in vitro*, NP incorporation into virus-like particles (VLPs), and replication of virus.

## Results

### Ensuring Tandem sdAbs Were Able to Bind Antigen

**Figure 2:**
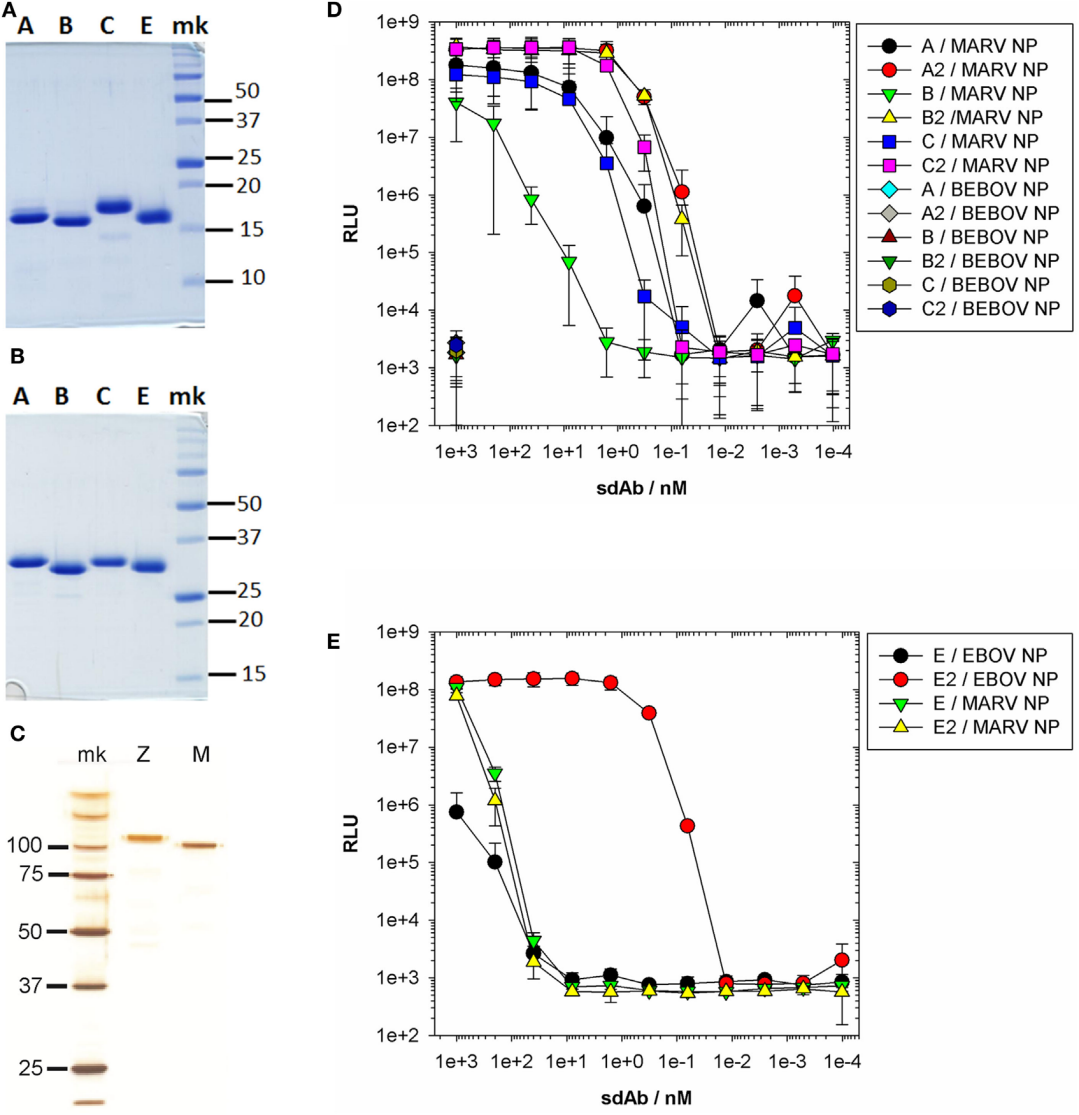
Purification of single-domain antibody (sdAb) proteins and recombinant nucleoprotein (NP) for ELISA characterization. Coomassie stained SDS-PAGE gel showing 5 µg of each sdAb monomer **(A)** and dimer **(B)** purified from *Escherichia coli* periplasm. **(C)** Silver stained SDS-PAGE gel of *Marburgvirus* (MARV) (M) and *Ebolavirus* (EBOV) (Z) NP preparations following large-scale transient transfection and purification through centrifugation steps and banding on CsCl gradients. **(D)** ELISA titration of the anti-MARV sdAb monomers and dimers over MARV NP with the highest concentration also applied to Bundibugyo NP (this was expressed at higher levels than Zaire NP and so was convenient to use for controls yet shares high homology at the C-terminal domain for our studies). **(E)** ELISA titration of anti-EBOV sdAb E monomer and dimer over EBOV NP and MARV NP.

Since all of our sdAb were originally selected using g3p phage display with free N-termini we had to ensure that fusions retained binding and ideally showed enhanced activity reflective of avidity when expressed as sdAb–sdAb dimers. We therefore assembled monomers and dimers of anti-MARV sdAb A, B, and C and anti-EBOV sdAb E in our standard dual expression and display vector pecan126 (16) for production of protein in the *Escherichia coli* periplasm. All clones were well expressed as monomers as expected (Figure 2A) and as dimers (Figure 2B) highlighting the modularity of the single-domain fold for recombinant expression campaigns. To test the binding ability of the sdAb *in vitro*, we generated polymeric MARV and EBOV NP by HEK 293T transient expression and a purification regime that ended with CsCl gradient centrifugation to band the polymers. Both MARV and EBOV proteins are highly pure with the monomeric versions revealed by gel analysis of boiled and reduced samples (Figure 2C). Titration of the anti-MARV sdAb on NP revealed that all of the dimeric forms were more effective at binding MARV NP than monomeric sdAb and showed no increase in cross-reactivity with EBOV NP at 1 µM concentrations of antibody (Figure 2D). However, the degree of improvement varied between clones with sdAb A and C showing modest 5-fold improvements, while sdAb B was around 500-fold resulting in dimeric sdAb A and B having equivalent binding potency.

Titration of the anti-EBOV sdAb E showed an almost 4-log improvement of the dimer over the monomer (Figure 2E), yet we are very guarded in assigning just dimerization as the sole cause of this shift since the background signal on MARV NP was unusually high at 1–0.1 µM antibody concentrations for both monomer and dimer. In our hands, sdAb E performs very well in sandwich-based detection of both viral and recombinant NP and is more conformationally sensitive than other anti-EBOV NP sdAbs (15). Antigens are well known to be unfolded when passively immobilized on plastic surfaces and this may have resulted in sdAb E monomer being a poor performer. We also know that sdAb E monomer can be sparingly soluble at high concentrations while the dimer does not show this problem, which would impact available binding in this form of titration. The combination of solubility and conformational issues may well account for sdAb E dimer performing so much better than monomer.

### Identifying Productive Anti-MARV and Anti-EBOV Intrabody Monomers and Dimers

**Figure 3:**
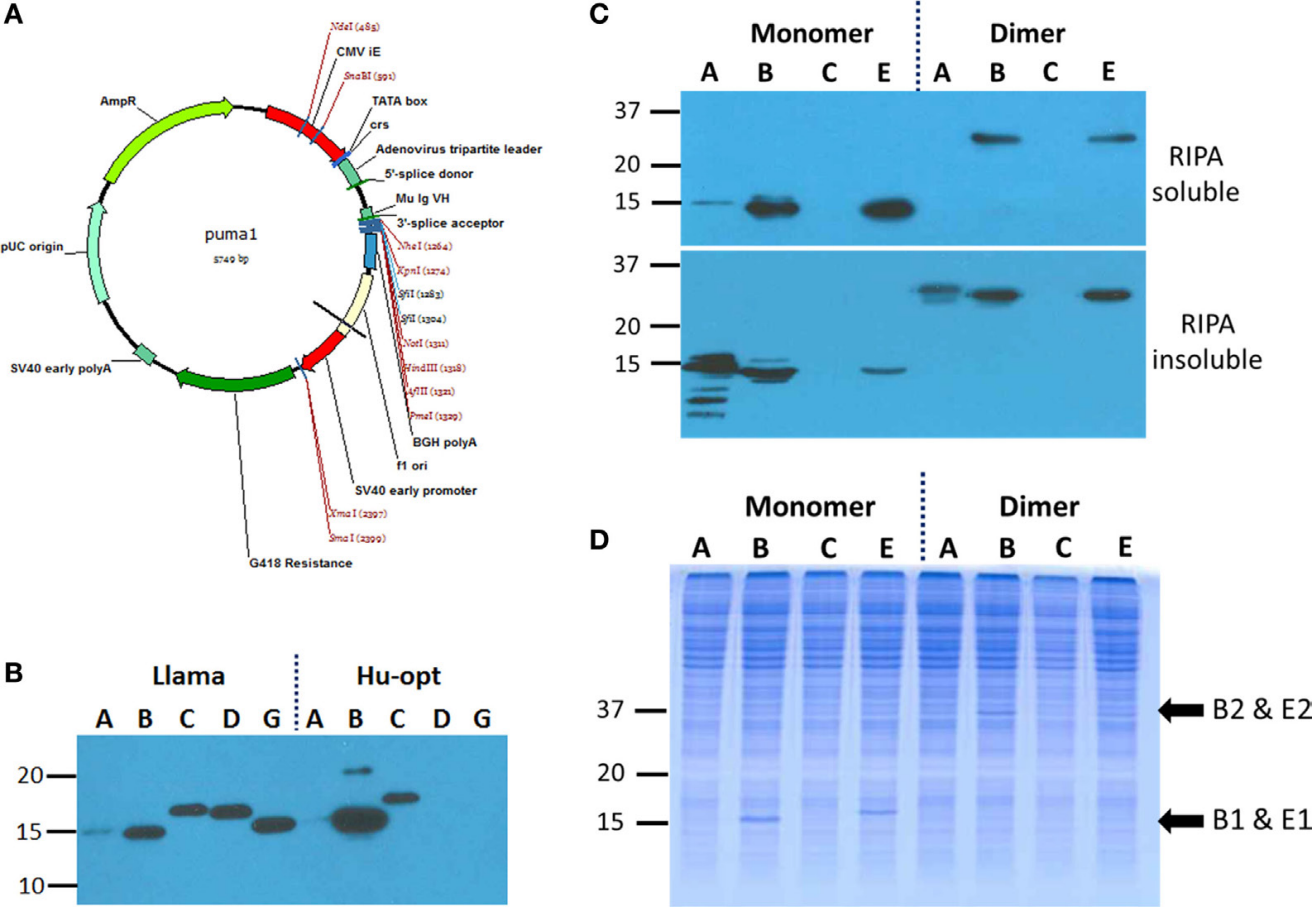
Evaluating expression of various single-domain antibody (sdAb) formats as intrabodies within HEK 293T cells. **(A)** Mammalian expression vector puma1 built for these studies utilizes a human cytomegalovirus immediate early gene enhancer and promoter (CMV IE) and the adenovirus tripartite 5′-non-coding region with a hybrid splice donor acceptor with other components from the pcDNA stable (Invitrogen) including high copy number in both *Escherichia coli* and HEK 293T cells and G418 resistance cassette for stable line generation. **(B)** Anti-C9 Western blot of whole cell lysates of HEK 293T cells transiently transfected with C9 tagged wild-type llama sdAb sequences or human optimized sequences identify those clones with low and high relative expression levels. Cells were harvested 72 h post-transfection. **(C)** Anti-C9 Western blot of HEK 293T soluble (including cytosol) and insoluble (including membranes) fractions following transient expression of llama sdAb as monomeric and dimeric versions. **(D)** Coomassie blue stained SDS-PAGE of the soluble fractions reveal visible bands at the expected places for sdAb B and E monomers and just visible are the corresponding dimers.

An enhanced mammalian expression vector was assembled to leverage the potent adenovirus tripartite sequence and hybrid intron for high level RNA processing and accumulation as noted by others (24, 25), yet leaving the convenient unidirectional *Sfi*I polylinker intact (Figure 3A). Our three primary anti-MARV NP candidate sdAb (A, B, and C), a fourth clone that expressed poorly in *E. coli* (D) and an EBOV NP specific clone (G) were first inserted into the pcDNASfi construct as both llama genes and human codon optimized genes. Plasmids were transfected into human embryonic kidney cells (HEK 293T) for transient expression and subsequent analysis by Western blotting of the whole cell extracts with sdAb detection through the C-terminal C9 tag. We were surprised to see the llama genes appeared to be more productive than the human optimized genes for all clones except sdAb B (Figure 3B). Monomeric and dimeric forms of the candidate genes were then assembled in puma1 and we used transient transfection and partial cell fractionation to determine the relative solubility of the monomers and dimers. Here, we switched from the EBOV-specific clone sdAb G to the highly cross-reactive clone sdAb E since our long-term goal is to develop countermeasures capable of broad reactivity among the Ebola species. Fortunately, both anti-MARV sdAb B and anti-EBOV sdAb E are produced at detectable levels in the soluble fraction as dimers (Figure 3C), though the levels appear somewhat reduced compared to monomers, perhaps reflecting the additional complexity required to fold tandem sdAb within the mammalian cytosol. Despite the apparent drop in expression levels, the dimeric forms of sdAb B and sdAb E are still just visible on Coomassie blue staining of the soluble fraction (Figure 3D) indicating that at least when expressed alone in the HEK 293T system, these sdAb formats are highly productive. We elected to take these two sdAb clones forward for further study in puma1 and for simplicity refer to them subsequently as M1 (anti-MARV NP sdAb B monomer), M2 (anti-MARV NP sdAb B dimer), E1 (anti-EBOV NP sdAb E monomer), and E2 (anti-EBOV NP sdAb E dimer).

### Examining NP Crosslinking Capacity of sdAb Monomers and Dimers *In Vitro*

**Figure 4:**
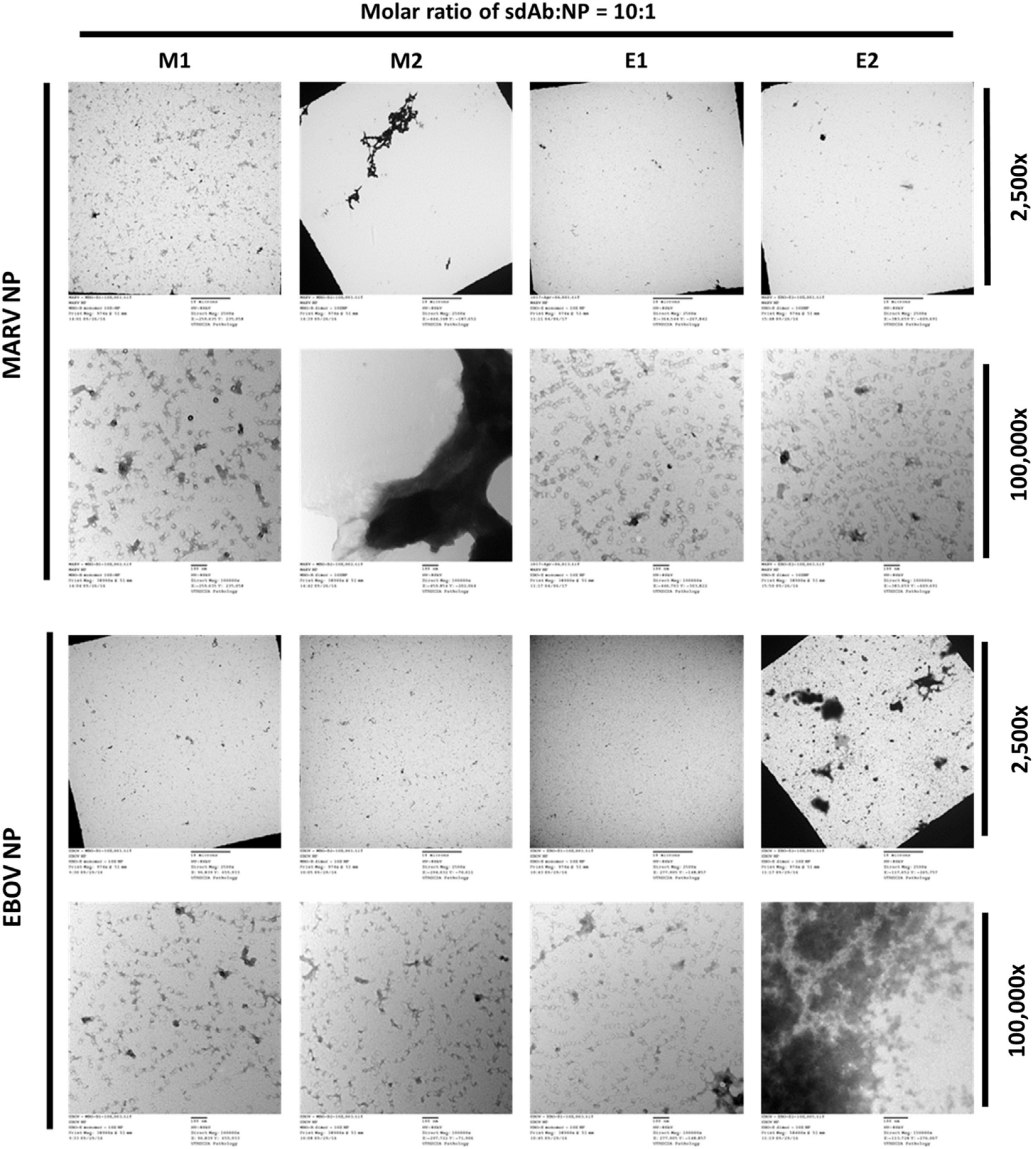
Examining the ability of single-domain antibody (sdAb) monomers and dimers to crosslink nucleoprotein (NP) *in vitro* by electron microscopy. 10:1 M ratios of the anti-*Marburgvirus* (MARV) monomer (M1) or dimer (M2), or anti-*Ebolavirus* (EBOV) monomer (E1) or dimer (E2) were combined with MARV or EBOV NP and equilibrated for 1 h prior to transmission microscopy. In the absence of crosslinking the individual helical filaments of NP, polymers are visible in the 100,000× images for both MARV and EBOV NP.

**Figure 5:**
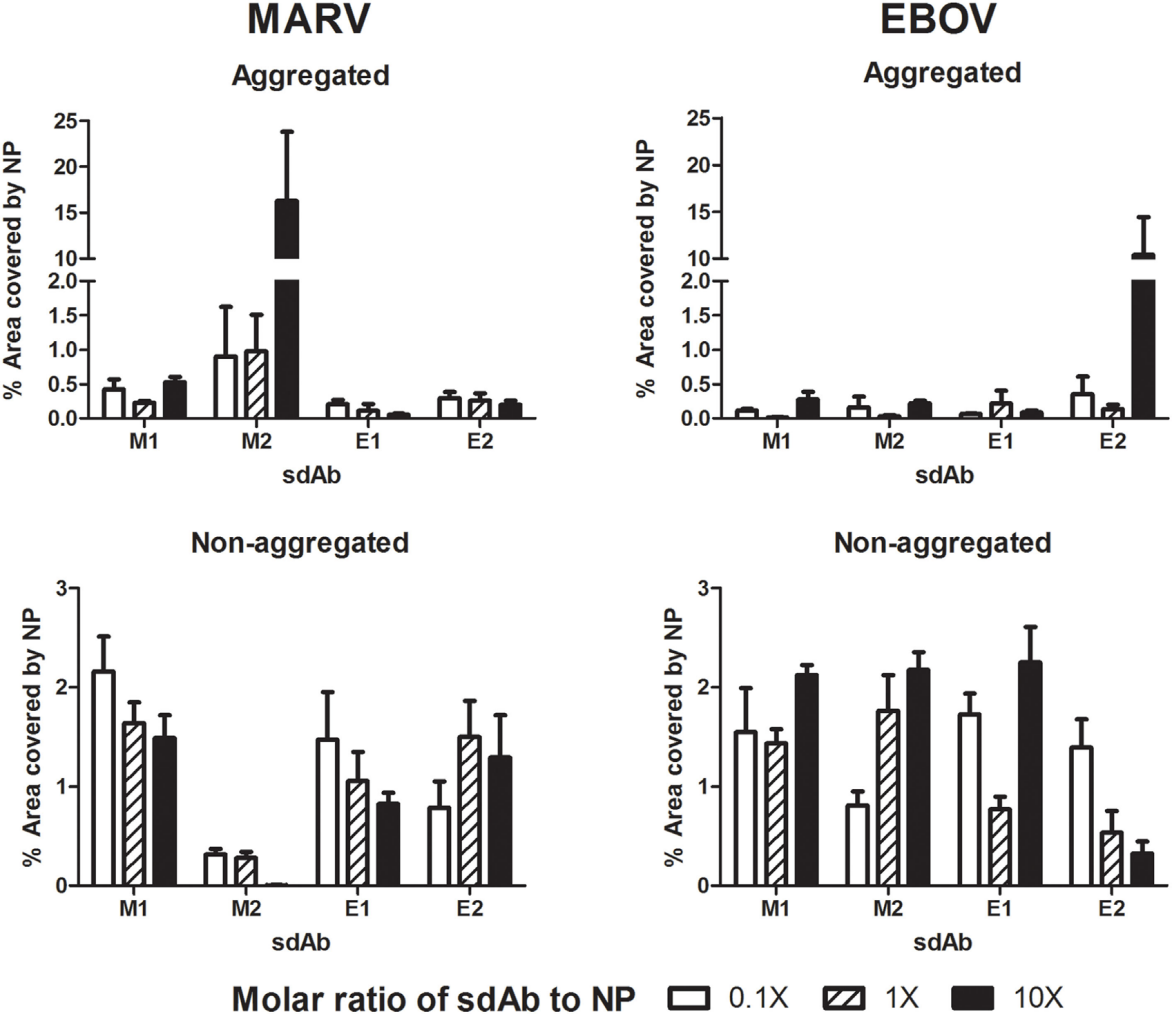
Quantification of the 100,000× images from Figure 4 using Cell Profiler to reveal the extent of aggregated and non-aggregated nucleoprotein (NP).

As we moved toward the intracellular immunofluorescent studies below, we conferred our sdAb and NP constructs with specific tags for visualization and so used these derivatives for *in vitro* crosslinking studies also. The M1, M2, E1, and E2 sdAbs were produced in *E. coli* with the C9 tag through periplasmic expression, IMAC purification, and gel filtration to generate highly pure proteins similar to those produced above (Figure S1A in Supplementary Material). Again, in multiple hands, we noticed similar production levels apparent for monomers and dimers from *E. coli* expressions suggesting tandem dimers are straightforward to produce in this host. Polymeric HA-tagged MARV and HA-tagged EBOV NP proteins were generated using the same optimized scaled up transient HEK 293T expression and purification protocol as above (Figure S1B in Supplementary Material). Different molar ratios of sdAb monomer and dimer proteins were combined with purified HA-NP, equilibrated for 1 h, and then the mixtures analyzed by transmission electron microscopy (Figure 4; Figure S2 in Supplementary Material). Here, the purity of the HA-NP preparations can be clearly seen as they form the classical helical filamentous structures seen by others. Only the dimeric forms of each sdAb were able to crosslink their corresponding HA-NP polymers in a genus-specific manner while the monomeric forms had no discernible effect. The crosslinking ability of the dimers extended to 1:1 and 0.1:1 M ratios of sdAb to HA-NP though uncrosslinked helical NP filaments are visible at lower ratios, suggesting the sdAbs are being out-titrated. At the lower concentrations, the anti-MARV dimer appeared to be better at crosslinking than the anti-EBOV dimer and following quantification, we assessed it to be approximately 10-fold more potent at aggregating HA-NP (Figure 5). Crosslinking NP polymers *in vitro* in this manner reassures us that the arbitrarily chosen 20 mer linker between the tandem sdAb B proteins does not restrict binding to two epitopes on a single polymer (intra-NP binding) but allows inter-NP binding. It would be of interest to explore whether alterations in linker length and composition might further improve crosslinking efficacy.

### Impact of sdAb Monomers and Dimers on VLP Formation

**Figure 6:**
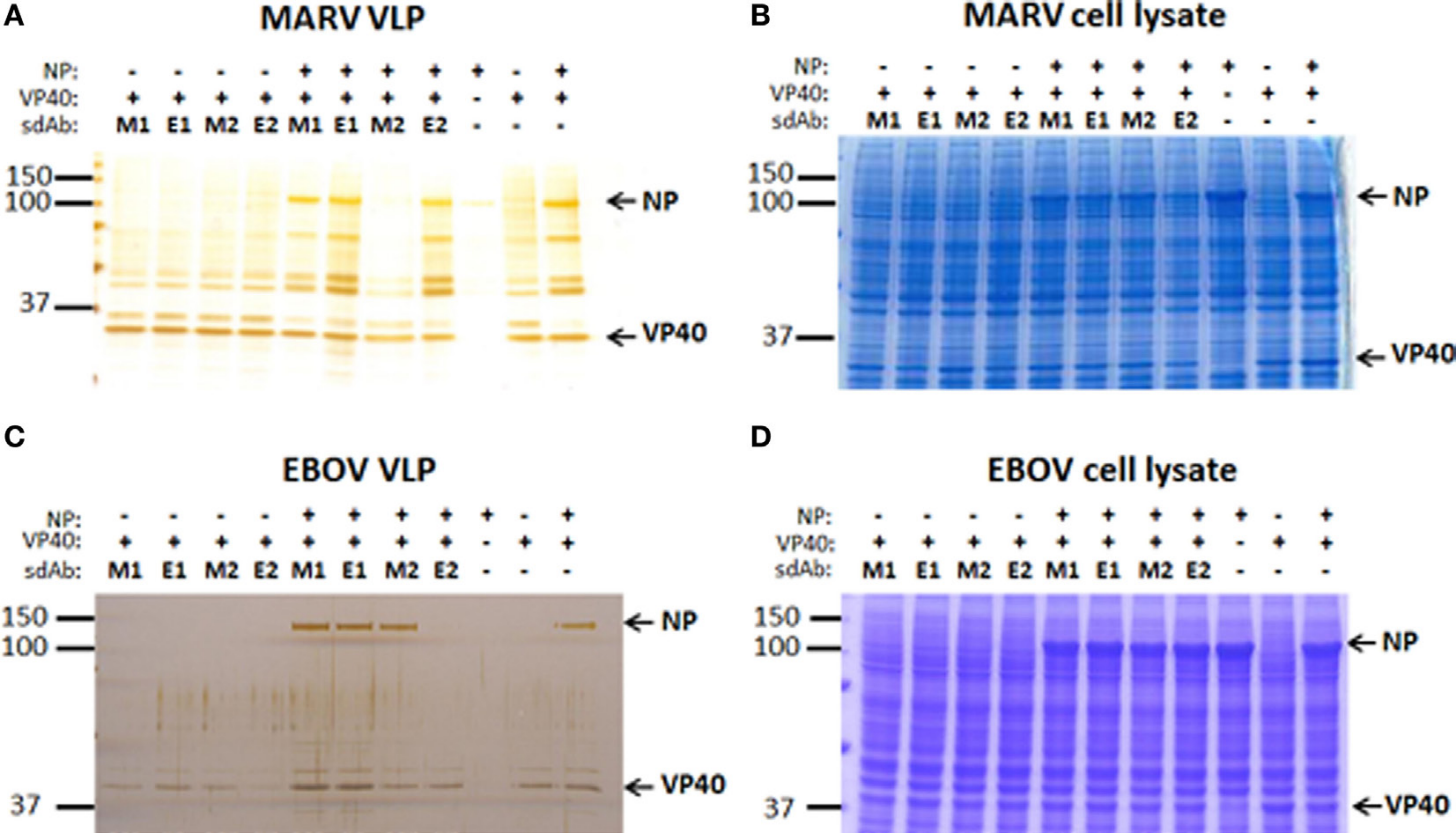
Exploring the impact of coexpressing the anti-*Marburgvirus* (MARV) single-domain antibody (sdAb) monomer (M1) or dimer (M2), or anti-*Ebolavirus* (EBOV) monomer (E1) or dimer (E2) on nucleoprotein (NP) incorporation into VLP. **(A)** Sliver stained SDS-PAGE analysis of crude VLP preparations following coexpression of MARV NP and/or VP40 with the various sdAb monomers and dimers. **(B)** Coomassie stained SDS-PAGE analysis of cell lysates stemming from the VLP production in panel **(A)**. **(C)** Sliver stained SDS-PAGE analysis of crude VLP preparations following coexpression of EBOV NP and/or VP40 with the various sdAb monomers and dimers. **(D)** Coomassie stained SDS-PAGE analysis of cell lysates stemming from the VLP production in panel **(C)**.

For both MARV and EBOV, VP40 expression alone is able to form enveloped VLPs (26, 27) and recombinant NP expression alone is enough to drive the formation of cytoplasmic inclusions (28, 29). When NP and VP40 are coexpressed, the result is VLPs made of NP and VP40. VLP composition in terms of ±NP therefore gives us a convenient way of analyzing the impact of Xintrabody expression on NP packaging. Since the particles are non-infectious, the approach is useable at BSL-2 for added convenience. Transient expression of combinations of MARV VP40, HA-NP, and the various sdAb constructs in HEK 293T cells revealed that NP was missing from those VLPs secreted from cells cotransfected with dimeric anti-MARV sdAb (M2) plasmid (Figure 6A). Examination of the cell lysates corresponding to these VLP expressions (Figure 6B) revealed that HA-NP was well expressed suggesting that the Xintrabody had not blocked NP production but a later step in VLP assembly. Importantly, the levels of sdAb are so low as to be not visible by Coomassie blue staining of SDS-PAGE gels when coexpressed with the viral proteins, yet the dimeric sdAb still has a dramatic inhibitory effect. A similar pattern for EBOV was evident when combinations of EBOV VP40, HA-NP, and the various sdAb were employed (Figure 6C) in that dimeric anti-EBOV sdAb (E2) inhibited NP incorporation into particles while other sdAb did not. Again, analysis of whole cell lysates revealed that EBOV HA-NP was being produced within the cells in large amounts suggesting a post-translational block of NP incorporation into VLPs was being elicited by E2 (Figure 6D). Figures S3A,B in Supplementary Material show Western blots of the MARV and EBOV cell lysates, respectively, to confirm expression of the sdAb monomers and dimers (since we were unable to visualize these through gel staining) and also confirm expression of NP and VP40.

### Impact of sdAb Monomers and Dimers on the Colocalization of VP40 and NP in Vero Cells

**Figure 7:**
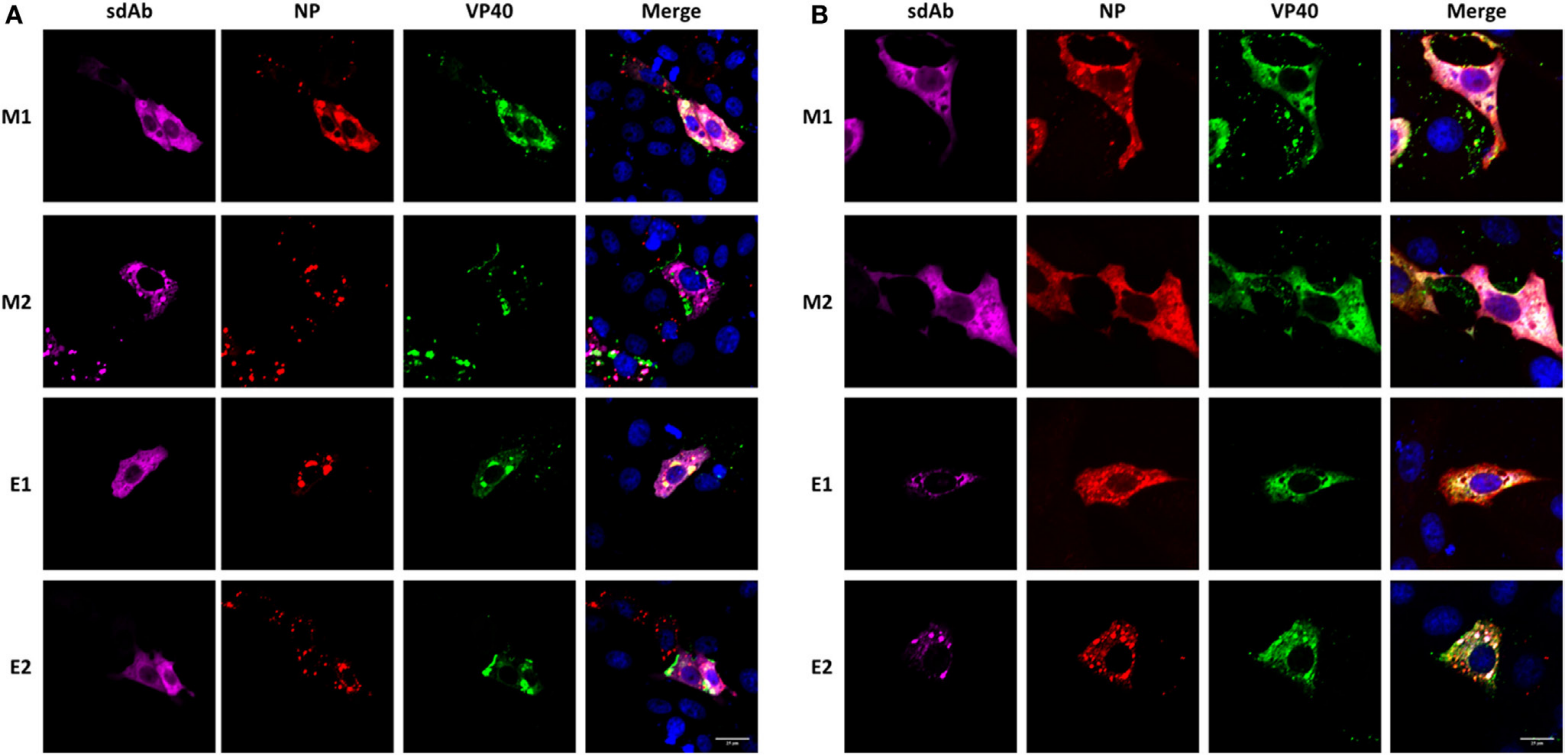
Localization of single-domain antibody (sdAb) monomer or dimer when coexpressed with nucleoprotein (NP) and VP40 and the impact on NP-VP40 colocalization. Immunofluorescence microscopy of transiently cotransfected Vero E6 cells producing anti-*Marburgvirus* (MARV) sdAb monomer (M1) or dimer (M2), or anti-*Ebolavirus* (EBOV) sdAb monomer (E1) or dimer (E2) with either **(A)** MARV VP40 and MARV NP genes or **(B)** EBOV VP40 and NP genes.

While HEK 293T cells are highly transfectable (in our hands >90%) and productive following transfection with SV40 origin containing plasmids they are poor cells for fine-scale microscopy and so here we employed Vero E6 cells which are naturally permissive for both MARV and EBOV replication. By analyzing the transient coexpression of MARV VP40, MARV HA-NP, and the M1, M2, E1 and E2 sdAb *via* immuno-staining the cells, we were able to monitor the impact of intrabody expression on NP and VP40 colocalization, which is normally evident during virion assembly. The M1 monomer shows a mix of cytosolic diffuse presence and localization with HA-NP as expected and does not appear to reduce the overlap of HA-NP with VP40 seen in the merged image (Figure 7A). In contrast, the M2 dimer appears to lose the diffuse cytosolic staining pattern, colocalizing at HA-NP pools, which are reflective of the virogenic inclusions. HA-NP VP40 colocalization appears to be inhibited and VP40 localization to the membrane appears reduced. As our ELISA data showed the dimeric M2 sdAb may well have better ability to target NP polymers over the monomeric sdAb and may well be enhanced at the NP pooling sites. It is important to note that in the presence of M2 dimer the HA-NP does not appear to be present at high levels in any locations other than focused punctate sites suggesting the dimeric sdAb is indeed restricting NP leaving to traffic to the membrane for assembly. The control anti-EBOV E1 and E2 as expected do not appear to colocalize with NP since they are not cross-reactive with MARV, enabling VP40 and NP to colocalize as seen in the merged images. We applied the same methodology to transient transfections of EBOV VP40 and HA-NP and saw the same general trends in that cognate dimer sdAb E2 localized to EBOV HA-NP pools and appeared to restrict colocalization with VP40, whereas the other antibody formats including the monomer sdAb E1 did not (Figure 7B).

### Impact of sdAb Dimers on MARV and EBOV Replication

**Figure 8:**
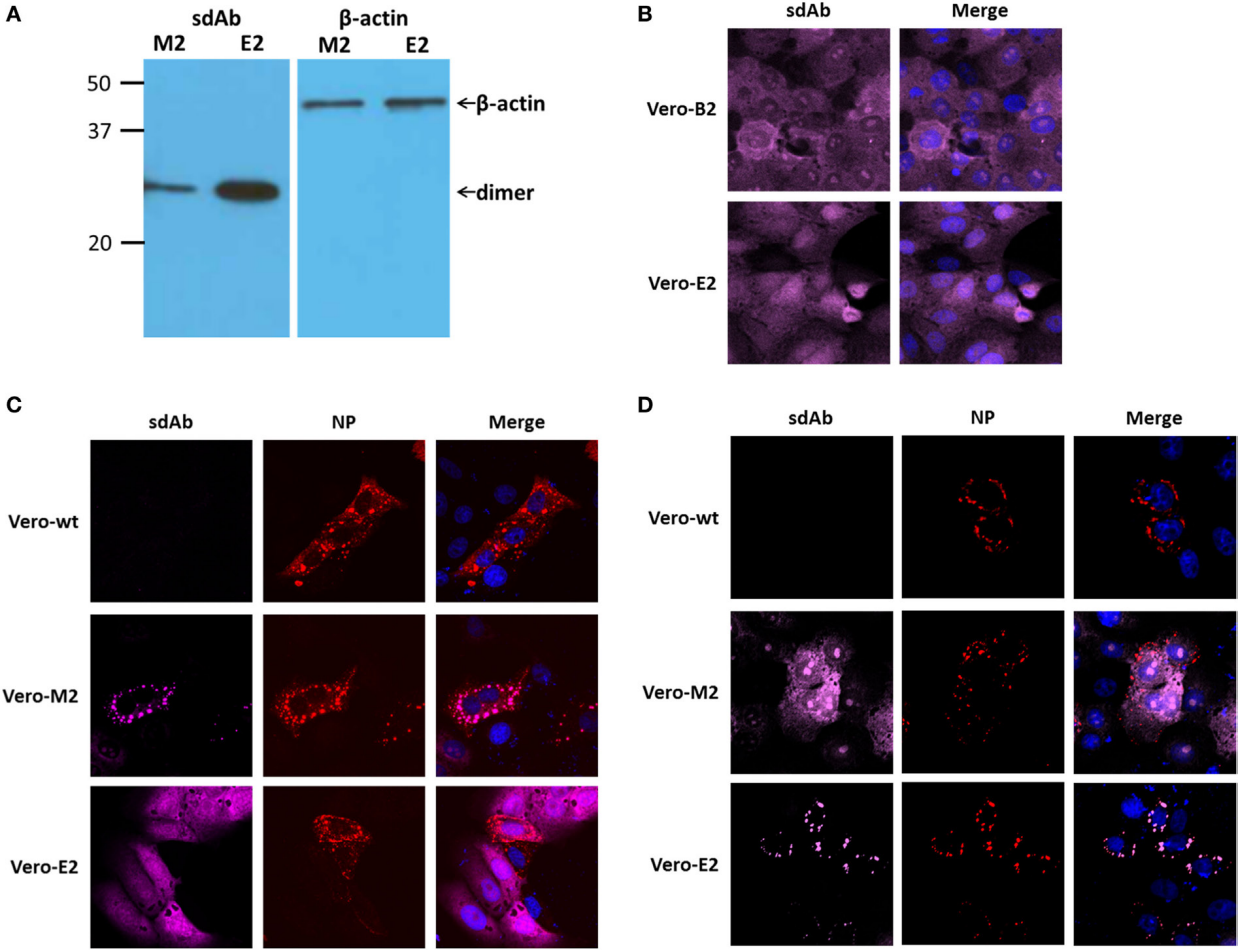
Production and distribution of single-domain antibody (sdAb) dimers within the Vero E6 based stable cell lines. **(A)** Western blot of stable cell lines expressing the anti-*Marburgvirus* (MARV) dimer (Vero-M2) or anti-*Ebolavirus* (EBOV) dimer (Vero-E2) and probing for antibody *via* the C9 tag or for β-actin. **(B)** Staining of parent (Vero-wt) and stable cell lines Vero-M2 and Vero-E2 for the distribution of antibody *via* the C9 tag. **(C)** Transient expression of MARV HA-NP within the parental and transgenic cell lines demonstrates the ability of the anti-MARV dimer but not the anti-EBOV dimer to colocalize with nucleoprotein (NP) puncta. **(D)** Transient expression of EBOV HA-NP within the parental and transgenic cell lines demonstrates the ability of the anti-EBOV dimer but not the anti-MARV dimer to colocalize with NP puncta.

The low (in our hands ≤5%) transfection efficiency of Vero E6 cells makes studying the direct impact of transient intrabody expression on viral replication very difficult since only a small number of cells will be producing recombinant protein. We therefore generated G418 selectable stable lines of the anti-MARV M2 (Vero-M2) and anti-EBOV E2 (Vero-E2) sdAb dimers in the puma1 expression vector and chose the cells having the highest levels of expression of products of the desired size by Western blot (Figure 8A) and generally diffuse staining when probed for the sdAb dimer C9 tag (Figure 8B). We confirmed the ability of each sdAb dimer to colocalize with its cognate NP following transient expression of either MARV HA-NP (Figure 8C) or EBOV HA-NP (Figure 8D) within parental, Vero-M2 or Vero-E2 cell lines.

**Figure 9:**
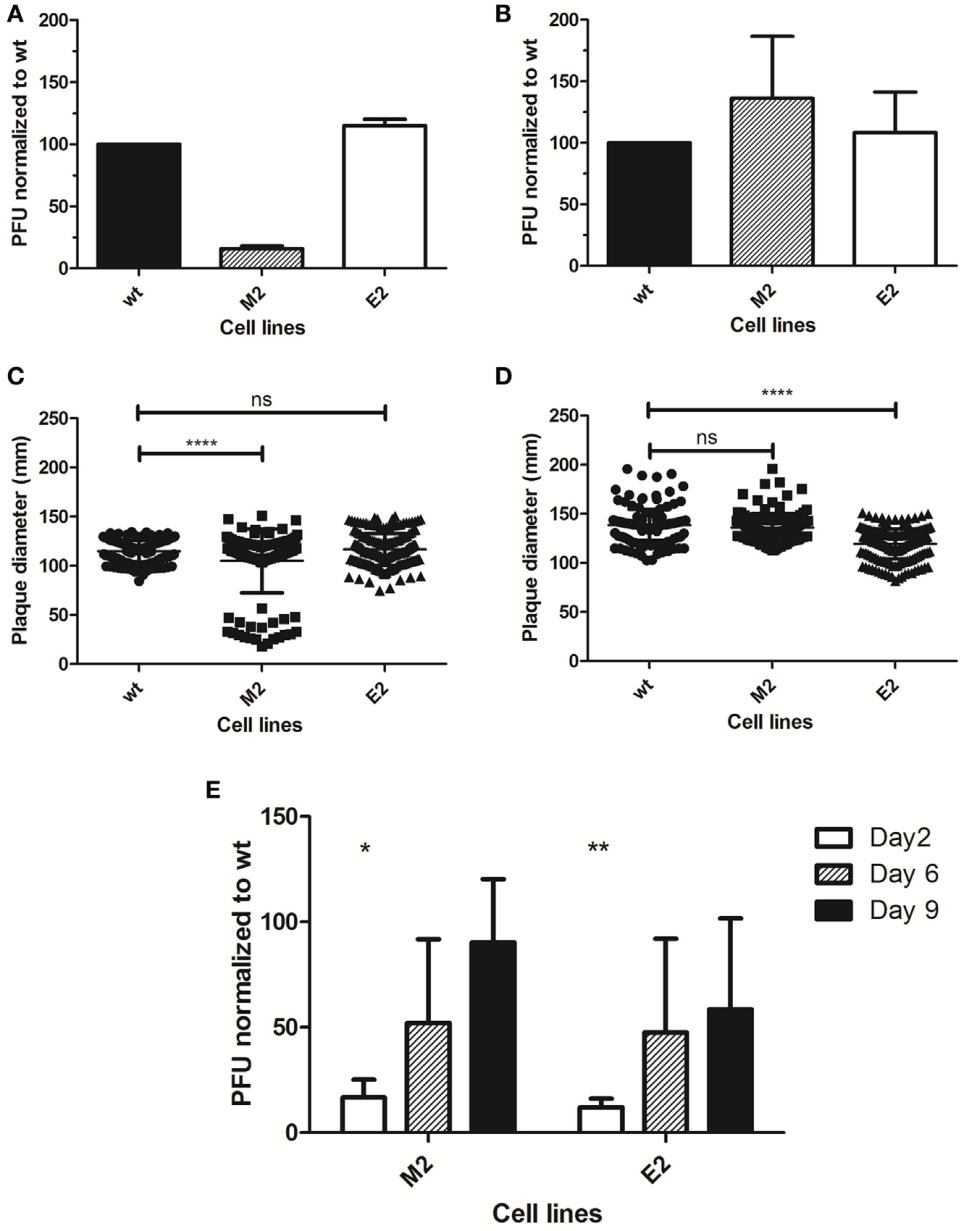
Impact of Xintrabodies on viral replication. *Marburgvirus* (MARV) **(A)** and *Ebolavirus* (EBOV) **(B)** were titrated on wild-type Vero cells (wt) or the stable lines expressing the anti-MARV dimer (M2) or anti-EBOV dimer (E2) and plaque formation under semi-solid overlay allowed to proceed for 10 days. The experiment was performed twice for MARV and three times for EBOV and error bars represent the SD. Diameters of approximately 150 plaques from the titrations were measured using ImageJ measure analysis tool and plotted for MARV **(C)** and EBOV **(D)**. An unpaired, one-tail *t*-test analysis revealed a *p*-value of <0.0001 (****) while ns indicates no significant difference. Bars indicate SD and the mean. **(E)** MARV and EBOV were used to infect wild-type or cognate single-domain antibody dimer stable cell lines in a growth kinetics format with liquid supernatant collections at the times indicated. Plaque titrations of the supernatants were performed on wild-type cells and results are graphed as a percentage of titers resulting from the wild-type time-course. The experiment was performed three times for MARV and twice for EBOV. An unpaired, one-tail *t*-test was conducted using Excel, * indicating a *p*-value <0.05 and ** indicating a *p*-value <0.005.

We next challenged the cells in a plaque titration with MARV to reveal that the Vero-M2 line is capable of reducing the number of plaques approximately fivefold when compared to control Vero wild-type cells or the Vero-E2 cell line (Figure 9A). When a similar experiment was performed with EBOV no difference in the number of plaques was observed between Vero-E2 and the other cell lines (Figure 9B), suggesting that plaque number was not being limited in the semi-solid overlay system. However, analysis of the plaque diameters revealed a significant decrease in the sizes of plaques for MARV in Vero-M2 (Figure 9C) and for EBOV in Vero-E2 (Figure 9D) compared to the non-cognate and wild-type cell lines. To better study the dynamics of viral replication and the impact of sdAb dimer upon it, we challenged the wild-type and cognate dimer cell lines within a liquid overlay setting. Supernatants were harvested in a time-course and the resulting progeny were titrated on wild-type cells. For both MARV and EBOV, there is a significant reduction in progeny virus early in the time-course but the impact diminishes as the experiment progresses (Figure 9E).

The impact of anti-MARV and anti-EBOV sdAb dimers are far from optimal and by no means providing sterilizing immunity, yet combined with all of the previous data are indicating that the Xintrabody approach has potential.

## Discussion

Herein, we sought to explore the potential for tandem sdAb dimers to promote aberrant interactions between viral components within the cytoplasm and focused on viral targets that we hypothesized would have the greatest impact owing to their polyvalent nature. In this manner, we take advantage of small amounts of dimeric sdAbs having a big effect on large amounts of antigen. The approach is admirably suited to targeting viral factories where large amounts of viral antigens can lie in close proximity and become very vulnerable to a crosslinking intrabody or Xintrabody approach. We certainly benefited from a panel of four anti-MARV sdAb clones from which to identify productive intrabodies. In the first place, perhaps tandemization is not ideal where free N-termini are required to take full advantage of bivalency and other N-terminal free oligomerization strategies such as leucine zippers (30) could be useful here. Second, several sdAbs were not especially productive when expressed in the cytosol, yet there are sequence tags available that can rescue poorly soluble clones (31) and can even enhance degradation of target antigens.

Our VLP analyses reveal an ablation of NP incorporation into particles despite large amounts of NP being produced and correspondingly minimal (not visible by Coomassie SDS-PAGE) amounts of Xintrabody indicating the promise of the approach. Microscopic analyses of dimeric sdAb revealed them to be focused on pools of NP and capable of restricting the distribution of NP to the areas where VP40 was localized. Combined with the electron microscopic views of *in vitro* crosslinking of purified NP and sdAb dimer preparations, it is tempting to speculate that in cells we are crosslinking RNC in the virogenic inclusions, yet finer resolution immuno-electron microscopy would be required to confirm this as certain. The stable cell lines expressing the anti-MARV and anti-EBOV Xintrabodies did not show the same dramatic effect as the VLP system but nonetheless resulted in smaller plaques and reduced virus yields when compared to wild-type cells at early time-points. Yet, when one considers the low levels of constitutively expressed recombinant proteins in cell lines not employing gene amplification or locus control regions, it is quite remarkable that our dimeric sdAbs had any impact at all. Furthermore, it is more than likely that host gene expression patterns are altered upon virus infection to reduce dimer production even further during the course of viral replication one could envisage employing a host cell promoter induced upon infection to drive more Xintrabody expression (32).

Antiviral sdAbs reactive with several viral glycoproteins have been shown to be massively improved in neutralization potency and breadth of reactivity when multimerized and can even protect against disease in animal models [for review, see Ref. (33)]. To the best of our knowledge, leveraging a multimerization routine for intrabodies has not yet been demonstrated and indicates Xintrabodies to be a novel potential therapeutic antibody development route. Though novel for recombinant intrabody technology, the strategy of crosslinking pathogen antigens as a defense mechanism is not new to nature. The immune system encodes natural crosslinkers of viral proteins including the carbohydrate-binding innate defensins, which can crosslink surface glycoproteins to create an inflexible barricade that inhibits entry to cells (34). The innate Mx proteins can be induced cytoplasmically and smother intracellular polymeric target proteins including many viral NPs (35). Adaptive immunity can also generate potent crosslinkers of monomers within the HA trimers of influenza A virus (36) to inhibit viral entry processes. Intra-spike crosslinkers based on IgG-derived Fab have also been further engineered to elevate anti-HIV potency a 100-fold and to reduce the chances of immune evasion (37). Heterologous lectins from algae such as Griffithsin also mediate crosslinking of viral glycoproteins and are being explored as antivirals against HIV (38) and can be enhanced by engineering multimerized forms (39). Perhaps for us the most relevant example of the potency of the crosslinking strategy is the anti-influenza A virus small compound drug nucleozin which exerts both early and late effects through impedance of N (NP) and RNC trafficking with the latter impact restricting packaging also (40, 41).

A common problem with antiviral strategies, including antibodies, small molecules, and even the innate Mx system, is the emergence of resistance within a viral population. Indeed, the anti-influenza VHH isolated recently using antiviral selection was shown to bind an epitope that was poorly conserved and liable to escape rapidly (42). Anti-VSV VHH monomers recently isolated using a similar approach to the anti-influenza A clones were also shown to generate viral escape mutants quite easily, again suggesting a pliable epitope (43). Our sdAbs are known to target highly conserved regions of MARV and EBOV NP lying at the C-terminus which are less likely to mutate to escape sdAb binding. It is tempting to speculate that along the lines of the Bjorkman study that dimerization of the sdAb will further enhance their immune constriction (38).

Although intrabodies are a good starting point to identify and evaluate antiviral sdAb formulations, they likely need transitioning to a protein delivery mechanism for long-term utility approaching the clinic. Here, we note that despite recombinant antibody technology having thrived for over 25 years, examples of success in assembling functional transbodies have been very limited (44). The approach may be fickle owing to incompatibility between compartments required for optimal antibody expression (periplasm) and the highly structured and charged tags required for transduction, e.g., HIV tat domain (45). Once protein is generated, the efficiency of reaching the cytosol may also be low due to endosomal entrapment though newer approaches appear to overcome this impasse using cell surface binding motifs to drive locally higher concentrations favorable for endosomal uptake and release (46). More recently, cyclic arginine rich motifs have shown promise as efficient sdAb delivery motifs (47) though these assemblies required *in vitro* ligation of the motif to the antibody, which may complicate scale-up. Despite these caveats, one group has pioneered monomeric transbodies to several viruses including influenza A (48), HCV (49), and more recently even Ebola (50) which is encouraging us to pursue the transition of Xintrabodies to Xtransbodies.

Multiple lines of evidence suggest that sdAbs have huge potential as therapeutics when delivered as proteins [see Ref. (51) for review] though they must be specifically tailored for their intended purpose. Their small size ensures rapid clearance *via* the kidneys and fast tissue penetration making them superior temporal imaging reagents but for applications requiring longer half-lives they require fusion to Fc or serum albumin-binding motifs. On the flipside, fast clearance should minimize the likelihood of human anti-llama antibody responses though this still can occur and appears to be on case-by-case basis related to the specific sdAb being used, the doses employed, and the disease under study (52). Since dimeric sdAbs are larger than monomeric sdAb they may have higher half-lives than expected and may well provoke host anti-sdAb antibody responses, though this may be outpaced by Xtransbody uptake. Erring on the side of caution, strategies for the humanization of pre-existing camelid sdAb such as our anti-MARV and anti-EBOV clones using CDR grafting to a universal humanized sdAb scaffold appearing very promising (53).

## Materials and Methods

### General Methods

Recombinant DNA methods were according to established procedures and employed commercially available reagents; Phusion High-Fidelity DNA Polymerase (Thermo Fisher Scientific, Waltham, MA, USA) was used for PCR amplification unless otherwise noted; restriction enzymes and β-agarase (New England BioLabs, Beverly, MA, USA); T4 DNA ligase, CIP and T4 PNK (Roche, Nutley, NJ, USA), GTG low-melting temperature agarose for in agarose cloning (Lonza, Walkersville, MD, USA); oligonucleotides (Integrated DNA Technologies, Coralville, IA, USA); synthetic human codon optimized sdAb and VP40 genes (Genscript, Piscataway, NJ, USA). Assemblies involving cloning and PCR amplification were sequenced through the inserts and junctions to verify the desired construct. Cloning was typically in XL1-Blue unless otherwise stated. Full details of cloning, oligonucleotides, maps, and sequences of resulting constructs are available on request. Parental sdAb genes employed in this work were anti-MARV NP sdAb A, B, C, and D with GenBank accession numbers MF780583, MF780584, MF780585, and MF780586, respectively; anti-EBOV NP sdAb E and G with GenBank accession numbers MF780602 and MF780604, respectively.

### Construction of Mammalian Cell Expression Vectors

C9-tagged sdAb A, B, C, D, and G genes were inserted into pcDNASfi as llama versions (GenBank accession numbers; MF871588, MF871589, MF871590, MF871591, and MF871592, respectively) or human codon optimized genes (GenBank accession numbers; MF871583, MF871584, MF871585, MF871586, and MF871587, respectively). pcDNASfi had its three *Nco*I sites deleted by Quick Change mutagenesis to form pcDNASfiNcoI^−^ with mutations verified by sequencing. Synthetic DNA representing a portion of the hCMV promoter, adenovirus tripartite leader, and hybrid murine splicing region [based on pMT2 (24)] with *Hin*dIII deleted from the intron was mobilized from pUC57 CMV-IVS *via Sna*BI and *Nhe*I to replace the resident 5′-ntr to form puma1. Monomeric and dimeric llama sdAbs with His_6_-C9 tags from pecan199 (see below) were mobilized to puma1 (GenBank accession numbers for monomeric sdAb A, B, C, and E are MF871599, MF871601, MF871603, and MF871605, respectively, while dimeric sdAbs are MF871600, MF871602, MF871604, and MF871606 respectively). Previous human codon optimized genes residing in pcDNASfi and encoding Marburg Musoke NP, Ebola Zaire Kikwit, and Bundibugyo NP (15) (GenBank accession numbers MF871598, MF871593, and MF871595, respectively) were subcloned to puma2 (pcDNASfiNcoI^−^ with the CMV intron A replacing the tripartite leader and murine splicing region) to generate ELISA substrates (see below). The Marburg and Zaire versions were also PCR amplified with a 5′-primer encoding an HA tag and inserted into puma2 to enable production of HA-tagged NP proteins for crosslinking and electron microscopy studies. Human codon optimized versions of Marburg Musoke and Ebola Zaire Kikwit VP40 genes (GenBank accession numbers MF871607 and MF871608, respectively) were also subcloned to puma2.

### Construction of Pecan199 *E. coli* Expression Vectors

Pecan73 is our standard tac promoter-driven *pel*B leader based sdAb expression vector (16) designed to secrete proteins to the periplasmic compartment and had a sdAb PCR amplified and re-inserted to replace the C-terminal His_6_ sequence with a C-terminal His_6_-C9 epitope tag (54) sequence to form pecan199.

### Construction of sdAb Dimers

sdAb genes were first mobilized from their original vectors (pecan21) (55) to enable soluble monomeric sdAb protein production of periplasmic hinge-less versions within pecan126 (16). Internal *Nco*I and *Pag*I sites within sdAb C were deleted by splice overlap extension PCR. Dimers were assembled by PCR amplifying the sdAb with an oligonucleotide encoding an *Nco*I site at the 5′-end to match the signal peptidase cleavage region and an oligonucleotide encoding a flexible (Gly_4_Ser)_4_ linker plus *Pag*I site at the 3′-end and re-inserting the amplicon into the cognate monomeric construct. Clones with the correct orientation were identified by restriction mapping with *Nco*I and *Not*I that released the dimer, while clones with the incorrect orientation would only release a monomer (GenBank accession numbers for pecan126 dimers sdAb A, B, C, and E are MF871575, MF871576, MF871577, and MF871578, respectively). SdAb genes were mobilized to pecan199 for expression for electron microscopic crosslinking studies (GenBank accession numbers: sdAb B monomer, MF871579; sdAb B dimer, MF871580; sdAb E monomer, MF871581; sdAb E dimer, MF871582).

### *E. coli* Expression of sdAb Monomers and Dimers

Pecan 126 or 199 sdAb constructs were mobilized to HBV88 or Tuner pRARE, respectively, and grown in 40 ml starter cultures of terrific broth (TB) plus 2% glucose at 30°C overnight with ampicillin (200 μg ml^−1^) and chloramphenicol (30 μg ml^−1^) in 250 ml baffled flasks (Bellco, Vineland, NJ, USA). Saturated cultures were transferred to 450 ml of fresh TB without glucose and shaken for 3 h at 25°C in 2,500 ml baffled flasks. Expression was induced by addition of IPTG to 1 mM for 3 h at 25°C, the cells pelleted (typical wet weights of 8–9 g) and osmotically shocked (56) by resuspension in 14 ml ice-cold 0.75 M sucrose in 100 mM Tris–HCl pH 7.5, addition of 1.4 ml of 1 mg ml^−1^ hen egg lysozyme (Sigma), followed by drop-wise addition of 28 ml of 1 mM EDTA pH 7.5 and swirling on ice for 15 min. A volume of 2.0 ml 0.5 M MgCl_2_ was added, swirling continued for 15 min, and cells pelleted. The 45-ml supernatant (osmotic shockate) was mixed with 5 ml of 10× IMAC (immobilized metal affinity chromatography buffer—0.2 M Na_2_HPO_4_, 5 M NaCl, 0.2 M imidazole, 1% Tween-20, pH 7.5), followed by 0.5 ml of High Performance Ni Sepharose (GE Healthcare) and the suspension gently mixed on ice for 1 h. Resin was pelleted at 3,000 rpm for 5 min (Beckman Allegra 6R swing out rotor) and washed twice with 40 ml of 1× IMAC buffer before elution with 2 ml of 500 mM UV grade imidazole in 1× IMAC buffer. Proteins were concentrated in Amicon 10 kDa ultrafiltration devices (Millipore, Billerica, MA, USA) to 200 µl for separation by gel filtration on a Superdex 200 Increase 10/300 GL column (GE Healthcare) operating in PBS. Proteins were quantified by UV adsorption and analyzed by SDS-PAGE and Coomassie blue staining for impurities.

### ELISA Titrations of sdAb Monomers and Dimers on NP Antigen

Antigen (NP) in 100 µl of PBS at 1 μg ml^−1^ was used to coat duplicate wells of high binding white ELISA plates overnight at 4°C. Plates were washed three times with PBS and each well probed with 100 µl of the antibody dilutions in PBS 2% non-fat Carnation milk (Nestlé, Vevey, Switzerland) for 1 h static. Probe was removed and plates washed three times with PBS 0.1% Tween-20 and two times with PBS. Anti-His_6_ HRP conjugate (Sigma) at 1 in 10,000 in PBS 2% non-fat milk was used to probe the wells for 1 h static. Signals were developed with injection of SuperSignal ELISA Pico Chemiluminescent Substrate (Thermo Fisher Scientific) using a luminometer (Turner BioSystems, Sunnyvale, CA, USA) and data collected with a 2 s integration. Duplicate wells of each dilution were averaged to derive a mean titration, the experiment repeated for an *n* of 2 with the final curves representing the mean of two experiments ± SD.

### Cell Lines

Vero E6 and HEK 293T cells were obtained from ATCC (Manassas, VA, USA). All cells were maintained in liquid nitrogen storage when not in use. Cells were maintained in Dulbecco’s modified Eagle’s medium (DMEM) with 4.5 g l^−1^ glucose, l-glutamine, and sodium pyruvate (Corning cellgro) plus 5% fetal bovine serum (Corning, NY, USA) and penicillin/streptomycin (complete medium) 37°C at 10% CO_2_ with humidity.

### Small-Scale Transient Recombinant Protein Expression in HEK 293 Cells

HEK 293T cells were seeded at 7.5e+5 cells per well in a 6-well plate in 3 ml of complete medium at 16–18 h prior to transfections. Constructs were transfected using previously established protocols (15, 57). Briefly, 30 µl DNA (approximately 45 ng μl^−1^) and 5 µl of linear polyethylenimine PEI (1 μg μl^−1^ pH 7) were combined and equilibrated for 10–15 min at room temperature in 300 µl serum-free DMEM prior to being carefully added to the cells. For experiments requiring coexpression of NP and VP40, 15 µl of each plasmid was used. For experiments requiring NP, VP40, and sdAb coexpression, 10 µl of each plasmid was used. Total DNA concentration was kept constant with use of an empty vector if required. At 24 h post-transfection, the medium was removed from cells and the monolayer washed with serum-free DMEM and further incubated with 2 ml of serum-free DMEM. At 72 h post-transfection, cells were washed gently with 1 ml warm PBS and then collected in 500 µl of collection buffer; for whole cell lysate collection, equal parts of Tris-buffered saline (TBS) and Laemmli sample buffer with reducing agent were used; for cell fractionation, radioimmunoprecipitation assay buffer (10 mM Tris–HCl pH 7.8, 150 mM NaCl, 1 mM EDTA, 1% NP-40) containing protease inhibitors (Roche Complete) was used. The soluble fraction was separated from the insoluble fraction by microcentrifugation at 15,000 rpm (5415D microcentrifuge, Eppendorf, Hauppauge, NY, USA) for 10 min at 4°C and collection of the supernatant. The pellet representing the insoluble fraction was resuspended in 500 µl of 1:1 TBS/Laemmli sample buffer. For crude VLP analysis, 2 ml of supernatant was collected and clarified in a microcentrifuge at 8,000 rpm for 5 min at room temperature. The supernatant was then overlaid on a 20% sucrose cushion and centrifuged at 38,000 rpm (Beckman SW55 rotor) for 2 h at 4°C. Crude VLPs were resuspended in 100 µl PBS. All samples were stored at −20°C before further processing.

### SDS-PAGE and Western Blotting

Samples were combined with an equal volume of Laemmli sample buffer if not already in a 1:1 mix and then heated at 100°C for 5 min. After cooling, samples were electrophoresed on appropriate percentage Laemmli gels. For silver staining, a SilverXpress Silver Staining Kit (Invitrogen) was used. For Coomassie blue staining, standard methods were used. For Western blotting, gels were semi-dry transferred to Immobilon P and the membrane blocked in 2% non-fat dried milk in PBS for 1 h prior to probing with either mouse monoclonal antibody RHO 1D4 (Flintbox, Chicago, IL, USA) specific for the C9 tag or mouse monoclonal antibody GT5512 (GeneTex, Irvine, CA, USA) specific for β-actin. For VP40 probing, mouse monoclonal 6B1 IgG_1_ (IBT 0203-016, IBT Bioservices, Rockville, MD, USA) was used for MARV VP40 while mouse monoclonal 3G5 IgG_1_ (IBT 0201-016) was used for EBOV VP40. HA-NP was probed for using a mouse IgG_2a_ (sc-7392, Santa Cruz Biotechnology, Inc., Dallas, TX, USA). Following washing three times with PBS 0.1% Tween-20 for 5 min and twice with PBS for 5 min, membranes were probed for 1 h with anti-mouse IgG (H + L) HRP (Thermo Fisher Scientific). Following further washing, the membrane was developed with SuperSignal West Pico Chemiluminescent substrate (Thermo Fisher Scientific) and images captured on CL-XPosure film (Thermo Fisher Scientific).

### Generating Vero E6 Cells Constitutively Expressing sdAb Dimers

Cells were seeded in two wells of a 6-well plate at 4e+5 cells per well 18 h prior to transfection. puma1 bearing the dimeric sdAbs M2 or E2 genes were linearized by *Ahd*I in the ampicillin resistance gene and DNA purified by phenol chloroform extraction and ethanol precipitation. 2.5 μg of DNA and 5 µg of PEI were combined and equilibrated for 15 min at room temperature in 300 µl serum-free DMEM and carefully added to the cells. At 24 h post-transfection, cells were trypsinized, like transfectants pooled and seeded into 10 15 cm diameter dishes. After 3 days, medium was changed and G418 added to 3.2 mg ml^−1^, which we had determined by titration to be the threshold for Vero E6 cell killing. When colonies were visible by eye they were trypsinized in cloning cylinders (Sigma) adhered to the plate with silicon grease (Beckman) and cells transferred to 24-well plates. Cell lines were analyzed by Western blotting and indirect fluorescence microscopy and one clone expressing dimeric E2 sdAb (Vero-E2) and one clone expressing dimeric M2 sdAb (Vero-M2) with the highest expression levels were used for further experimentation.

### Large-scale Transient Expression of NP and HA-NP in HEK 293T Cells

Cells were seeded in 16 10 cm dishes at 5e+6 cells per dish in 20 ml of complete DMEM 16–18 h prior to transfection. Per plate, 105 µl Qiagen miniprep puma2 NP or HA-NP DNA (100 ng μl^−1^) and 41 µl PEI were combined and equilibrated for 20 min at room temperature in 2.5 ml serum-free DMEM prior to being carefully added to the cells. Cells were collected 48 h post-transfection by trypsinisation in 4 ml trypsin–EDTA solution (Sigma) with two plates worth of cells combined into 50 ml Falcon tubes and topped up to 50 ml with PBS. Cells were pelleted at 1,000 rpm for 5 min (Beckman Allegra 6R swing out rotor) washed once with phosphate buffered saline and repelleted. The cells were lysed in 4 ml of ice-cold hypotonic buffer (20 mM HEPES pH 7.5, 5 mM KCl, 1.5 mM MgCl_2_, 1 mM DTT, 1 tablet EDTA-free protease inhibitors per 50 ml). DNA was sheared by passing through a 30-G needle several times on ice. Samples were microfuged in 2 ml tubes at 6,000 rpm for 10 min at 4°C and the supernatants transferred to fresh tubes and re-centrifuged at 13,000 rpm for 10 min. Clarified samples were pooled and concentrated in two 15 ml 100 kDa cutoff Amicon centrifugal filters at 3,500 rpm (Beckman Allegra 6R, swing out rotor, room temperature) until the volume was approximately 800 µl. Samples were clarified by microcentrifugation at high speed for 5 min immediately before loading 400 µl atop CsCl gradients (40–25%, 5% steps in TNE—10 mM Tris–HCl pH 7.4, 150 mM NaCl, 1 mM EDTA). Gradients were centrifuged at 25,000 rpm (Beckman SW41Ti) for 18 h at 20°C. The NP or HA-NP bands were collected by side-puncture with an 18-G needle; samples combined, and dialyzed in 10 kDa cutoff Slide-A-Lyzer cassettes (Thermo Fisher Scientific) against PBS at 4°C. Samples were analyzed by SDS-PAGE and silver staining (NP) or Coomassie blue staining (HA-NP) for purity, quantified by micro-BCA assay, and stored at 4°C until needed.

### Fluorescence Microscopy

Vero E6 cells were transfected on 8-well micro slides (ibidi, Fitchburg, WI, USA) as above with the following changes: cells were seeded at 1.5e+4 cells/well; 250 ng miniprep DNA and 500 ng PEI were mixed in 30 µl serum-free DMEM and added to cells. As before, total DNA was kept constant with an empty vector used if needed. At 48 h, slides were washed with warm serum-free DMEM twice. Slides were fixed with 10% formalin for 24 h at 4°C. Slides were washed three times with PBS before permeabilizing with 0.1% Triton X-100 (Sigma-Aldrich) in PBS for 10 min. Slides were washed three times with PBS and blocked with 3% BSA in PBS for 1 h. Slides were washed once with PBS and stained with appropriate primary antibody for 1 h in 1.5% BSA in PBS. Cells were washed three times with PBS and then probed with secondary antibodies for 1 h each in 1.5% BSA in PBS, followed by three washes. The primary antibodies used were chicken polyclonal anti-HA tag IgY (ab9111, abcam, Cambridge, MA, USA) at 1:500 for HA-NP detection; mouse monoclonal 6B1 IgG_1_ (IBT 0203-016) at 1:500 for MARV VP40 detection; mouse monoclonal 3G5 IgG_1_ (IBT 0201-016) at 1:1,000 for EBOV VP40 detection; RHO 1D4 conjugated to Alexa Fluor 647 (antibody labeling kit, Invitrogen) for sdAb detection *via* the C-terminal C9 tag. The secondary antibodies used were donkey anti-mouse IgG labeled with Alexa Fluor 488 (Invitrogen A21202); goat polyclonal anti-chicken IgY labeled with Alexa Fluor 594 (abcam ab150176). Sequential probing of the VP40 antigens was performed before the C9 antibody was applied to minimize anti-mouse secondary cross-reactivity. Slides were placed at 4°C for imaging at a later date and Hoechst stain (Invitrogen H33342) in PBS was added to each well for nuclear staining if required at room temperature for at least 20 min prior to microscopy. Slides were viewed using an Eclipse Ti confocal microscope (Nikon) and NIS Elements Imaging Software. For analysis, 10–20 fields were viewed using a Plan Apo VC 20× DIC N2 objective with a numerical aperture of 0.75 giving 0.62 µm per pixel. For images presented in this manuscript, the same objective was used along with a 5× zoom factor giving 0.12 µm per pixel. Images presented were representative of typical cells and protein distribution. ImageJ within Fiji was used to process *Z*-stack images with average intensity projections to obtain two-dimensional images. For optimal viewing of protein localization, the color balance was adjusted so that the intensity histogram covers only the signal.

### Electron Microscopy of HA-NP and sdAb Mixtures

Purified HA-NP (1 µM) was equilibrated with sdAb concentrations of 10, 1, and 0.1 µM binding sites in a final volume of 250 µl for 1 h static. The mixtures were then allowed to passively adhere to grids for 5 min and stained with 20 µl of uranyl acetate for 1–2 min. Images were taken on a JEOL JEM-1230 transmission electron microscope. At least eight representative fields were imaged. For quantitative analysis, Cell Profiler was used on the 100,000× magnified images. To determine the area covered by aggregated NP, objects greater than 12 pixels were accepted. To determine the area covered by helical NP, objects between 2 and 12 pixels were accepted. GraphPad Prism was used to graph results.

### Viral Growth Assays

Live virus work was performed within the full-suit BSL-4 laboratory at Texas Biomedical Research Institute, following all local and federal guidelines as part of the Select Agent Program. Marburg Musoke and Ebola Zaire Kikwit were amplified and titrated on Vero E6 cells as described previously (15). Vero E6 wild-type or the constitutive sdAb-expressing cell lines Vero-E2 and Vero-M2 were first used as plaque titrants by seeding 8e+5 cells per well in duplicate 6-well plates in 2 ml of complete medium approximately 18 h prior to infection. Medium was removed and 500 µl of virus in serum-free DMEM added to each duplicate well with serial dilutions (−2 to −6 with one no virus control well per plate). Plates were then incubated at 37°C with humidity and 10% CO_2_ with gentle rocking for 1 h. During incubation, aliquots of 1% SeaPlaque GTG agarose were heated to boiling and let cool to 37°C. Eagle’s MEM (Lonza) plus 4 mM l-glutamine and 2 mM sodium pyruvate was mixed 1:1 with agarose. Virus was carefully removed with a P1000 pipette and overlayed with 2 ml of the EMEM agarose. The agarose was allowed to solidify for 10 min at room temperature and the plates incubated at 37°C for 10–11 days with humidity and CO_2_. The plates and overlays were then fixed in 10% formalin for 24 h and removed from the BSL-4 *via* the chemical dunk tank. Overlays were then removed and cells stained with 1% crystal violet for plaque counting. The experiment was repeated a total of two times for MARV and three times for EBOV. Following scanning of the plates, approximately 150 clearly separated plaques for each cell line were analyzed using ImageJ to record the diameters. An unpaired one-tail *t*-test within Graphpad was used to identify statistical significance.

Marburg Musoke and Ebola Zaire Kikwit growth kinetics were evaluated on Vero E6 wild-type or the constitutive sdAb dimer expressing cell lines Vero-M2 and Vero-E2, respectively, seeded at 8e+5 cells per well in 6-well plates in 2 ml complete medium at approximately 18 h prior to infection. Medium was removed and 500 µl of virus in SF DMEM added per well at a multiplicity of infection of 0.01. Plates were incubated at 37°C with humidity and 10% CO_2_ with gentle rocking for 1 h. The virus was carefully removed with a P1000 pipette, cells washed once with 1 ml of complete medium, and incubated at 37°C for 2, 6, or 9 days in 2 ml complete medium. At time of collection, medium from a single well was removed by a P1000 pipette, clarified in a 2 ml Eppendorf tube in a microfuge at 8,000 rpm for 5 min at 4°C. The samples were then transferred to Sarstedt 2 ml screw cap micro tubes (Sarstedt, Nümbrecht, Germany) and immediately stored at −80°C until required. Each time point was titrated on duplicate wells of wild-type Vero E6 cells and the titer averaged. The time-course, collections, and titrations were repeated a total of three times for MARV and twice for EBOV with plaque forming units per milliliter obtained on each stable cell line presented as percentages of those obtained on the wild-type cells ±SD. An unpaired one-tail *t*-test was used to identify statistical significance.

## Author Contributions

TD, LS, and AH designed experiments, performed the work, and analyzed the data. AH wrote the paper.

## Conflict of Interest Statement

The authors declare that the research was conducted in the absence of any commercial or financial relationships that could be construed as a potential conflict of interest.
