# Supplementary material for: Intracellular Crosslinking of Filoviral Nucleoproteins with Xintrabodies Restricts Viral Packaging
Source: Front Immunol. 2017 Sep 27;8:1197. doi: 10.3389/fimmu.2017.01197 (PMC5623874; doi:10.3389/fimmu.2017.01197)
Supplement: Supplementary file 1 [file Presentation_1.pdf]

Supplementary Material

Intracellular Crosslinking of Filoviral Nucleoproteins with Xintrabodies Restricts Viral Packaging

Tamarand Lee Darling, Laura Jo Sherwood, Andrew Hayhurst*

*** Correspondence:** Corresponding Author: ahayhurst@TxBiomed.org

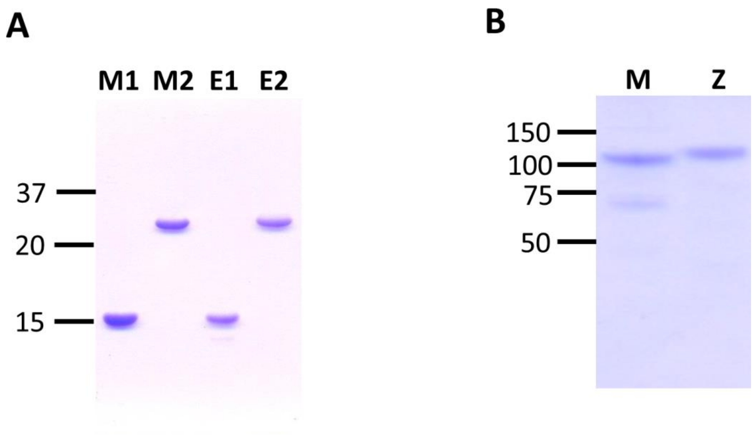

Supplementary Figure 1. Coomassie stained SDS-PAGE analyses of the sdAb and NP preparations employed in the *in vitro* crosslinking experiment. **A.** Purified monomeric anti-MARV (M1), monomeric anti-EBOV (E1), dimeric anti-MARV (M2) and dimeric anti-EBOV (E2) sdAb preparations derived from expression vector pecan199 which confers the C9-His$_6$ tag to the C-terminus. **B.** Purified recombinant HA-NP from MARV (M) and EBOV (Z) expression constructs.

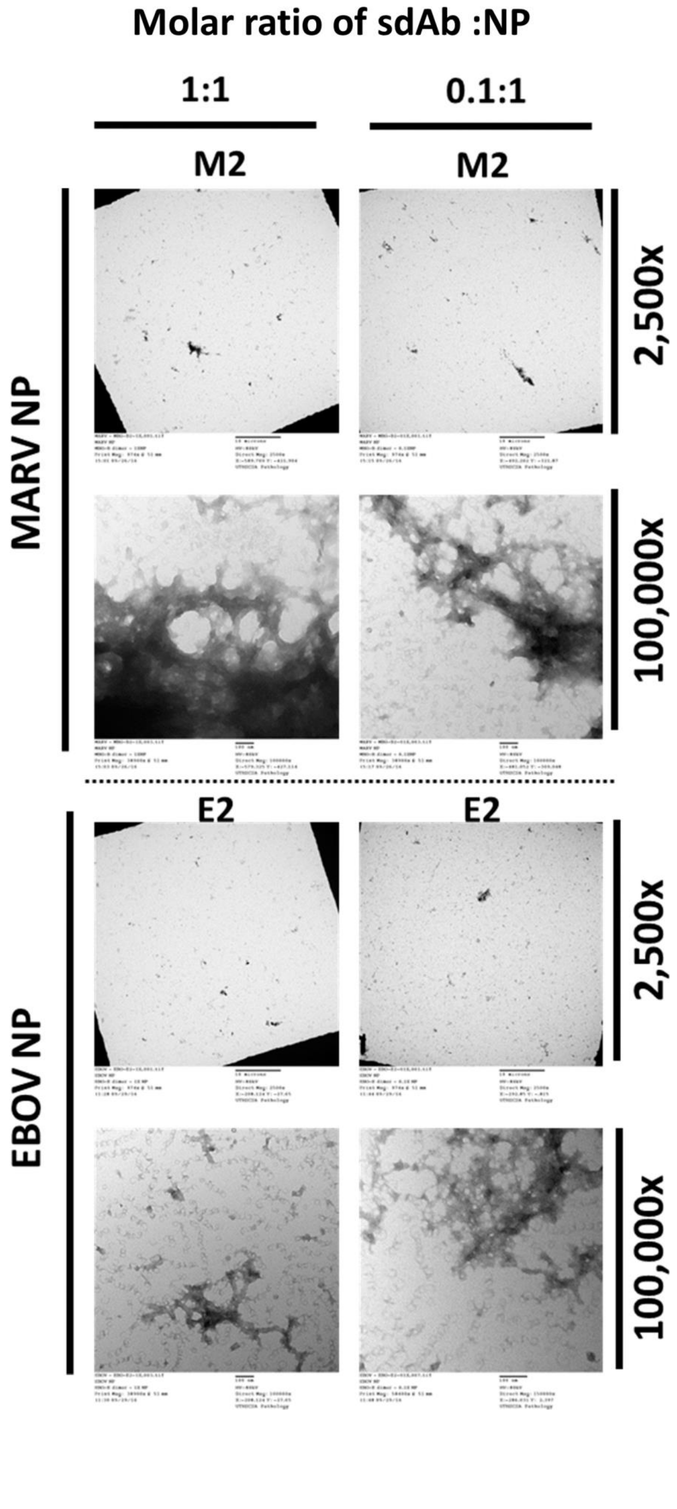

Supplementary Figure 2. Examining the ability of sdAb dimers to crosslink cognate NP *in vitro* at lower antibody:antigen ratios. Dimeric sdAb and NP mixtures at 1:1 and 0.1:1 molar ratios were assembled and allowed to equilibrate for 1 hour prior to transmission electron microscopy. Anti-MARV dimer (M2) was combined with MARV HA-NP while anti-EBOV dimer (E2) was combined with EBOV HA-NP.

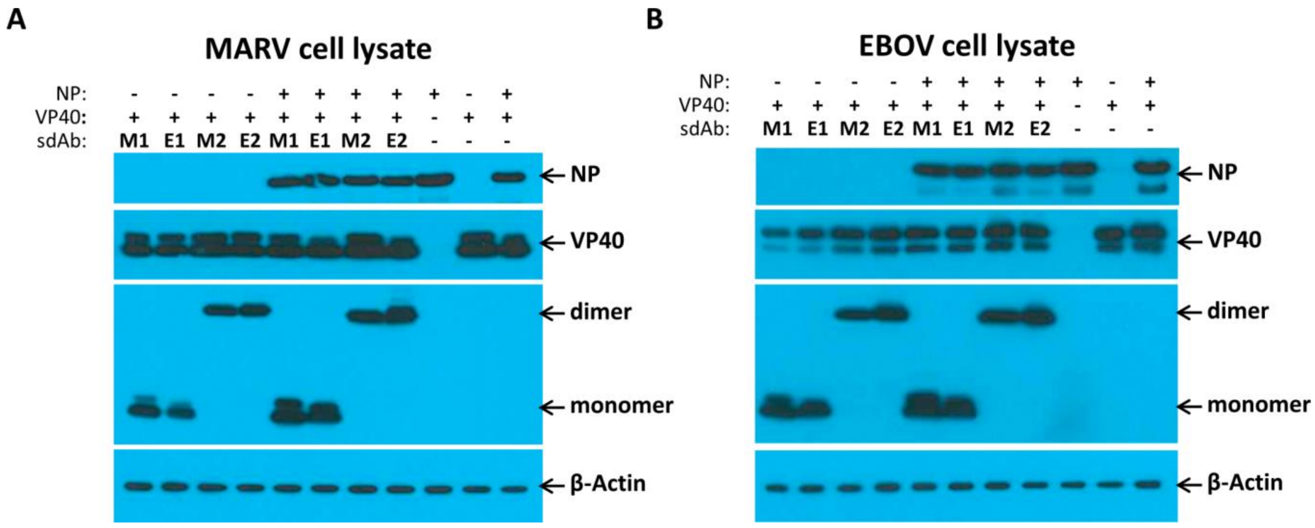

Supplementary Figure 3. Western blots of cell lysates used in exploring the impact of transiently coexpressing the anti-MARV sdAb monomer (M1) or dimer (M2), or anti-EBOV monomer (E1) or dimer (E2) on NP packaging. **A.** MARV lysates where the NP and VP40 are derived from MARV. **B.** EBOV lysates where the NP and VP40 are derived from EBOV. The sdAb constructs are indicated as monomers and dimers.
